# IL-2 Modulates TAMs Derived Exosomal MiRNAs to Ameliorate Hepatocellular Carcinoma Development and Progression

**DOI:** 10.1155/2022/3445350

**Published:** 2022-02-21

**Authors:** Hao Chen, Chao Tang, Chun Tan, Fei Wu, Zhenhan Li, Wenyan Ji, Linming Lu, Chongjun Xu, Zhengchao Shen, Yanqiang Huang

**Affiliations:** ^1^Research Center for the Prevention and Treatment of Drug Resistant Microbial Infecting, Youjiang Medical University for Nationalities, Baise 533000, China; ^2^Department of Pathology, Wannan Medical College, Wuhu 241002, China; ^3^Postdoctoral Research Station of Clinical Medicine, Jinan University, Guangzhou 510632, China; ^4^School of Medicine and Holistic Integrative Medicine, Nanjing University of Chinese Medicine, Nanjing 210023, China; ^5^Graduate School, Youjiang Medical University for Nationalities, Baise 533000, China; ^6^School of Clinical Medicine, Wannan Medical College, Wuhu 241002, China; ^7^Department of Ultrasound, The First Affiliated Hospital, Yijishan Hospital of Wannan Medical College, Wuhu 241002, China; ^8^Department of Hepatobiliary Surgery, The First Affiliated Hospital, Yijishan Hospital of Wannan Medical College, Wuhu 241001, China

## Abstract

*Background*. Interleukin-2 (IL-2) is proved to play an irreplaceable role in antitumor regulation in numerous experimental and clinical trials. Tumor-associated macrophages (TAMs) are able to release exosomes to promote the development and progression of hepatocellular carcinoma (HCC) as essential component of microenvironment. In this study, our intention is to explore the effects of the exosomes from TAMs with IL-2 treatment on HCC development. TAMs were collected and cultured from HCC tissues. The exosomes from the TAMs treated with IL-2 (Exo^IL2-TAM^) or not (Exo^TAM^) were identified and used to treat HCC cells *in vivo* and *in vitro*. The proliferation, apoptosis, and metastasis of HCC cells were measured. The changes of miRNAs in exosomes were explored to clarify the possible mechanisms. Both decrease of cell proliferation and metastasis and increase of apoptosis were observed with Exo^IL2-TAM^ treatment compared with Exo^TAM^*in vivo* and *in vitro*. miR-375 was obviously augmented in Exo^IL2-TAM^ and HCC cells treated with Exo^IL2-TAM^. Taken together, IL-2 may modulate exosomal miRNAs from TAMs to ameliorate hepatocellular carcinoma development. This study provides a new perspective to explain the mechanism by which IL-2 inhibits hepatocellular carcinoma and implies the potential clinical value of exosomal miRNAs released by TAMs.

## 1. Introduction

Primary hepatocellular carcinoma (HCC) is the third most common malignant tumor in cancer mortality [1]. The treatment of HCC is mainly surgical resection and liver transplantation [2]. Because of its high degree of malignancy, the five-year survival rate is still less than 50% and the recurrence rate is high. Recurrence and metastasis have become the biggest obstacle to improve the therapeutic effect and survival rate of HCC [3]. The occurrence, development, invasion, and metastasis of tumors are not only determined by the malignant tumor cells themselves but also closely related to the tumor microenvironment [4–6].

It has been proved that there are a large number of tumor-associated macrophages (TAMs) in tumor microenvironment. Recent studies have confirmed that TAMs are mainly M2 type macrophages, which can promote tumor growth by affecting angiogenesis, immunosuppression, invasion, and metastasis [7–9]. Exosomes are membrane vesicles with a diameter of 30–150 nm [10]. They can be secreted by the cells and widely exist in various body fluids. The exosomes carry RNA and protein components and are involved in signal transduction and immune escape of tumor as well as the diagnosis and treatment of a large number of diseases [11, 12]. Compared with adjacent normal tissues, the expression levels of miRNAs changed in many malignant tumors [1]. It has been reported that the exosomes derived from TAMs promoted HCC cell proliferation and metastasis [13–16].

IL-2 is a multifunctional cytokine in the immune system which effectively activates and enhances the phagocytosis and killing ability of macrophage [17, 18]. IL-2 has been used in lots of clinical trials for the treatment of HCC [2]. However, it remains unknown whether IL-2 regulates the exosomes released from TAMs. Given the emerging role of IL-2 in curing HCC and regulating the function of macrophages, this study is aimed to explore the effects of exosomes from IL-2-treated TAMs on HCC development and the possible mechanisms.

## 2. Materials and Methods

### 2.1. Cell Lines and Reagents

HepG2 and QJY‐7703 cells were purchased from Tongpai Biotechnology (Shanghai, China) and were maintained in Dulbecco's Modified Eagle Medium (DMEM, Wisent, CA, USA) containing 10% fetal bovine serum (FBS) (ExCell Bio, China). Recombinant human IL-2 was purchased from Sigma-Aldrich LLC.

### 2.2. Patients and Primary Human TAMs Isolation and Culture from Tissue Specimens

Primary human HCC specimens were collected from the patients who suffered from hepatectomy at Youjiang Medical College Affiliated Hospital. Patient's consent was obtained and the procedures were approved by the Ethics Committee of the Youjiang Medical College Affiliated Hospital. Human fresh tumor samples were minced with scissors. The macrophages were isolated and cultured by Percoll (GE Healthcare) density gradient centrifugation. TAMs were treated with IL-2 for 24 hours before the supernatants were collected. The treated concentration was 20 ng/ml.

### 2.3. Isolation and Labelling of Exosomes

Exosomes were isolated from the cell culture media with Total Exosome Isolation Reagent (Thermo Scientific) according to the manufacturer's instructions. The purified exosomes were then labelled with PKH26 (Umibio) according to the manufacturer's directions.

### 2.4. Exosomes and TAMs Cells Observed by Transmission Electron Microscopy

The samples were fixed with 2% glutaraldehyde and 2% paraformaldehyde in 0.1 mol/L sodium cacodylate buffer at pH 7.3 for 3 hours at room temperature. After air drying, samples were mounted on specimen stubs and visualized using transmission electron microscope.

### 2.5. RNA Isolation and qPCR

Total RNA was isolated from cells or mouse tissues using TRIzol reagent (Invitrogen), following the manufacturer's instructions. The RNA was then analyzed using real‐time qPCR with SYBR Green PCR Master Mix (Roche Applied Science, Mannheim, Germany).

### 2.6. Western Blot Analysis

Exosomes or cells were lysed in RIPA containing protease inhibitors. 20 *μ*g exosomes were separated by SDS‐PAGE and transferred to PVDF membranes (Millipore, Bedford, MA, USA). The membranes were then incubated with antibodies CD63 (1 : 1000; Abcam, Cambridge, MA, USA), Calnexin (1 : 1000; CST,USA), PCNA (1 : 1000; CST, USA), cyclin D1 (1 : 1000; CST, USA) E‐cadherin (1 : 1000; CST, USA), N‐cadherin (1 : 1000; CST, USA), Tubulin (1 : 5000, Abcam, Cambridge, UK), Bax (1 : 1000; CST,USA), Bcl-2 (1 : 1000; CST,USA), MMP-2 (1 : 1000, Proteintech, Chicago, Illinois, USA), and MMP-9 (1 : 1000, Proteintech, Chicago, Illinois, USA).

### 2.7. Quantification of Apoptosis by Flow Cytometry

Apoptosis was determined using an Annexin V-FITC/PI apoptosis detection kit (eBioscience, USA). The cells were assessed via flow cytometry.

### 2.8. EdU Assays

For EdU assays, HepG2 cells were added to 24-well plates, and, after 24 h of incubation with exosomes, *EdU* (Sigma-Aldrich) staining was conducted based on the protocols.

### 2.9. Migration Assay

Cell migration assays were conducted on 24-well Transwell cell culture chambers with 8 *μ*m sized pores (Corning, USA). HepG2 cells were suspended in 500 *μ*L of medium and added to the upper inserts. After 24 hours of incubation, the cells remaining in the upper chamber were removed, and the cells on the lower surface of the chamber were fixed with 4% paraformaldehyde and stained with 0.5% crystal violet.

### 2.10. Scratch Test

Scratch assays were performed to assess cell migration in vitro. First, HepG2 cells were seeded in 6‐well plates until a confluent monolayer was formed. Then, upon confluence, cells were scratched with a 10 *μ*L sterile pipette tip. Pictures of the scratch were then taken at different time points under the microscope. The cell migration rate was calculated as (width at 0 hours – width at 24 hours)/width at 0 hours.

### 2.11. Xenograft Mouse Models

All animal experiments were performed after obtaining the approval of the Animal Ethics Committee of Youjiang Medical University for Nationalities. QJY‐7703 cells in logarithmic growth phase were prepared into cell suspension. Axillary subcutaneous injection of cells (5 × 10^5^) of BALB/c nude mice (4–6 weeks old, *n* = 6 per group) was performed. After the mean tumor volume had grown to be palpable, PBS or the indicated exosomes were injected every 2 days for 7 times. Tumor volumes were measured after 10 days every 5 days until mice were sacrificed 30 days later. 

### 2.12. The Liver and Lung Metastasis Experiment

The 6–8-week-old nude mice were divided into three randomized groups (*n* = 12 per group), and QJY‐7703 (5 × 10^5^) was injected into the mice via tail vein with or with out the indicated exosomes. 30 days after cell injection, the mice were euthanized and were necropsied to assess the metastatic ability of HCC cells. The liver and lung tissues of mice were further examined by H&E staining.

### 2.13. TUNEL Staining

Apoptotic cells in tumor tissues were detected by TUNEL assay according to the standard procedure. After being fixed in 4% paraformaldehyde, the tissues were stained by 50 *μ*L TUNEL reaction mixture (Roche) for 60 min at 37°C. The cell nucleus was stained with DAPI and observed using the Olympus microscope.

### 2.14. Difference in Expression

The data came from the miRNAseq data of level 3 BCGSC miRNA Profiling in the LIHC (hepatocellular carcinoma) project of TCGA (https://portal.gdc.cancer.gov/). The software for analyzing unpaired sample data was R (version 3.6.3) (statistical analysis and visualization), the R package was ggplot2 [version 3.3.3] (for visualization), and the molecule was hsa-miR-375 (MIMAT0000728). There were a total of 425 cases in the unpaired sample. When analyzing the paired samples, the paired samples in the data (424 cases) were retained. At the same time, on the UALCAN website, we also showed the expression of hsa-miR-375 in different cancer stages. In order to show the expression level of miR-375 in different stages, we entered the target molecule on the UALCAN website and basically selected hepatocellular carcinoma to get the results.

### 2.15. Clinical Significance

The R packages for drawing survival curves were survminer package (version 0.4.9) (for visualization) and survival package (version 3.2-10) (for statistical analysis of survival data); the prognosis type was overall survival. The R packages for ROC curve drawing were pROC package (version 1.17.0.1) (for analysis) and ggplot2 package (version 3.3.3) (for visualization), and the clinical variables were Normal versus Tumor. Interpretation of ROC curve results were as follows: the abscissa was the false positive rate (FPR), and the ordinate was the true positive rate (TPR). The value of the area under the ROC curve was between 0.5 and 1. The closer the AUC was to 1, the better the diagnostic effect is. AUC is less accurate when it is from 0.5 to 0.7, and it has a certain accuracy when it was between 0.7 and 0.9. There is higher accuracy when AUC is above 0.9.

### 2.16. Correlation Scatter Plot

For correlation analysis, we also downloaded the level 3 HTSeq-FPKM format RNAseq data in the TCGA (https://portal.gdc.cancer.gov/) LIHC (hepatocellular carcinoma) project, which is the protein expression data. RNAseq data in FPKM (Fragments Per Kilobase per Million) format were converted to TPM (transcripts per million reads) format and log2 conversion was performed.

### 2.17. Statistical Analysis

Data were presented as mean ± SD. Comparisons between groups were performed using one-way ANOVA, and Tukey's procedure for multiple range tests was performed. All experiments for cell cultures were performed independently at least three times and in triplicate each time. *P* < 0.05 was considered to be significant. All analyses were performed with Graphpad Prism 7.0 and SPSS18.0.

## 3. Results

### 3.1. The Internalization of Exosomes by HCC Cells

**Figure 1:**
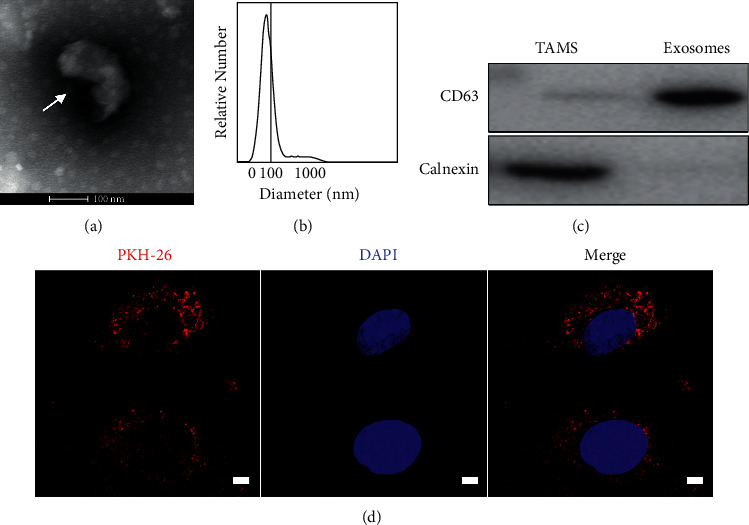
The exosomes identification and internalization in HCC cells. (a) Exosomes from TAMs observed under a transmission electron microscope. (b) NTA analysis of isolated exosomes; the *X*-axis represents the diameter of the vesicle and the *Y*-axis represents the number of vesicles. (c) CD63 and Calnexin expressions in TAMs and exosomes measured by western blot. (d) Internalization of PKH26-labelled exosomes (red) derived from TAMs by HepG2 cells.

To obtain exosomes, we collected HCC tissues to isolate and culture TAMs. The exosomes were isolated from macrophage supernatants with ultracentrifugation and were observed by transmission electron microscope (TEM) which were hemisphere with one side depression with typical characteristics (Figure 1(a)). The diameter of exosomes ranged from 30 to 150 nm, as shown in Figure 1(b). Western blot was used to further confirm that the isolated small spheres were exosomes by detecting CD63 and not Calnexin derived from TAMs (Figure 1(c)). To determine the effects of exosomes on HCC cells, we examined whether exosomes could be taken up by HCCs. An immunofluorescence assay was performed by using exosomes labelled with PKH26, a red fluorescence dye. Red fluorescence was clearly observed in HCC cells around nuclei using the confocal microscope (Figure 1(d)).

### 3.2. The Effects of the Exosomes on HCC Development and Progression In Vitro

**Figure 2:**
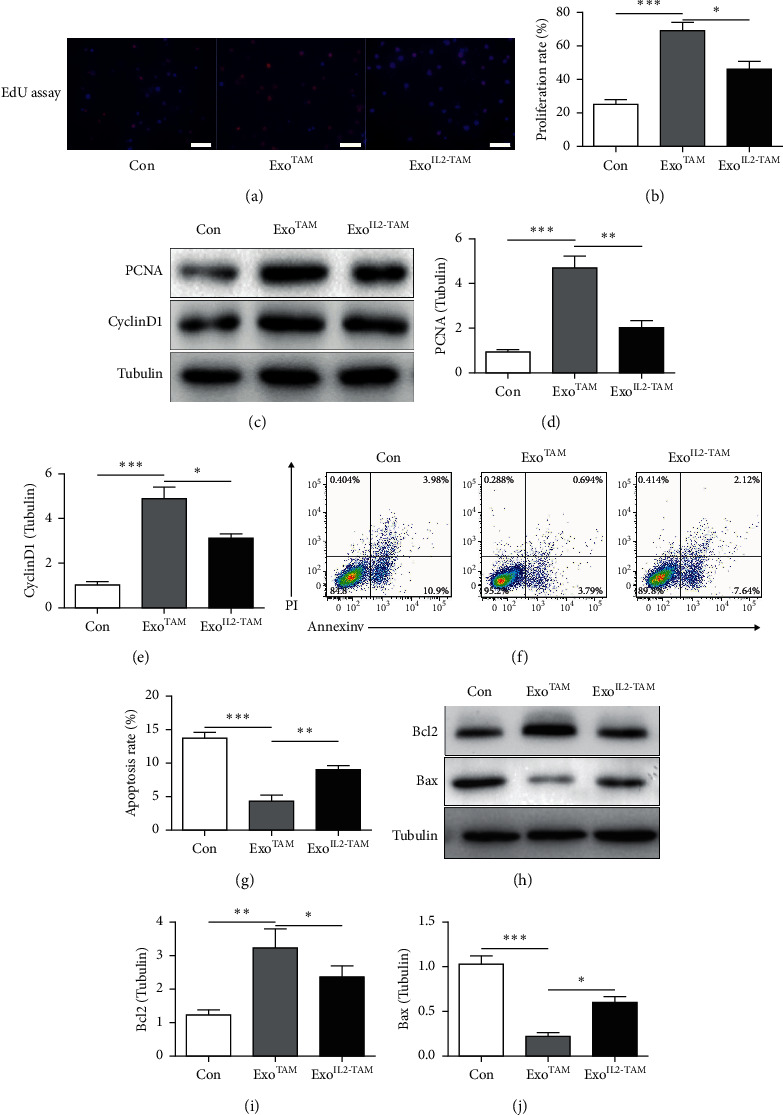
The effects of the exosomes on HCC cell proliferation and apoptosis. (a) Representative immunostaining images of EdU^+^ cells *in vitro.* (b) Percentages of EdU^+^ nuclei over total number of nuclei. (c–e) Western blot analysis of PCNA and cyclin D1 expression. (f–g) Flow cytometry for Annexin V-APC and propidium iodide (PI) staining in cells treated with exosomes for 24 h. The representative plots (f) and quantification (g) are shown. (h–j) Western blot analysis of Bax and Bcl-2 expression. Error bars, SD. ^*∗*^*P* < 0.05; ^*∗∗*^*P* < 0.01; and ^*∗∗∗*^*P* < 0.001.

First, the effects of the exosomes on the proliferation of HepG2 cells were explored. The results revealed that, when compared with Con group, the cell proliferation abilities were increased in the Exo^TAM^ group. These effects were ameliorated in the Exo^IL2-TAM^ group compared with the Exo^TAM^ group (Figures 2(a)–2(e)). Then, the effects of the exosomes on the apoptosis of HepG2 cells were explored with flow cytometry. HCC cells' apoptotic rate was decreased in the Exo^TAM^ group companied with Con group. Exo^IL2-TAM^ partly reversed these effects compared with the Exo^TAM^ group (Figures 2(f) and 2(g)). In line with these findings, the protein levels of the antiapoptosis molecule Bcl-2 increased and the proapoptosis molecule Bax decreased in the Exo^TAM^ group compared with Con group, and these changes were partly reversed in the Exo^IL2-TAM^ group (Figures 2(h)–2(j)).

**Figure 3:**
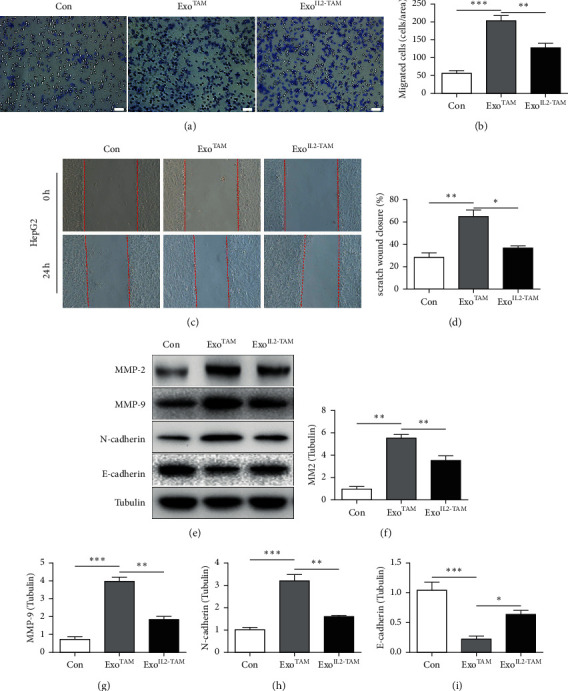
The effects of the exosomes on HCC cell migration *in vitro.* (a) Representative photographs of migration cells in Transwell coculture system. Scale bar = 100 *μ*m. (b) Qualification of migration cells in Transwell coculture system. (c) Representative photographs of migration cells in scratch test. (d) Qualification of migration cells in scratch test. (e–i) Western blot analysis of MMP2, MMP9, N-cadherin, and E-cadherin expression. Error bars, SD. ^*∗*^*P* < 0.05; ^*∗∗*^*P* < 0.01; and ^*∗∗∗*^*P* < 0.001.

Last, the effects of the exosomes on the migration of HepG2 cells were measured. Transwell assay and scratch test were used to explore whether IL-2 treatment affects TAMs-derived exosomes to influence the migration abilities of HepG2 cells. The cell migration abilities were increased in the Exo^TAM^ group compared to Con group; these abilities were reduced in the Exo^IL2-TAM^ group compared to the Exo^TAM^ group (Figures 3(a)–3(d)). Western blot analysis also revealed that the activation of EMT by the Exo^TAM^ treatment was restored in the Exo^IL2-TAM^ group (Figures 3(e)–3(i)).

Collectively, our findings indicated that the effects of exosomes from TAMs promoting proliferative and migration behaviors and inhibiting apoptosis were partly reversed by IL-2 treatment *in vitro*.

### 3.3. The Effects of the Exosomes on HCC Development and Progression In Vivo

**Figure 4:**
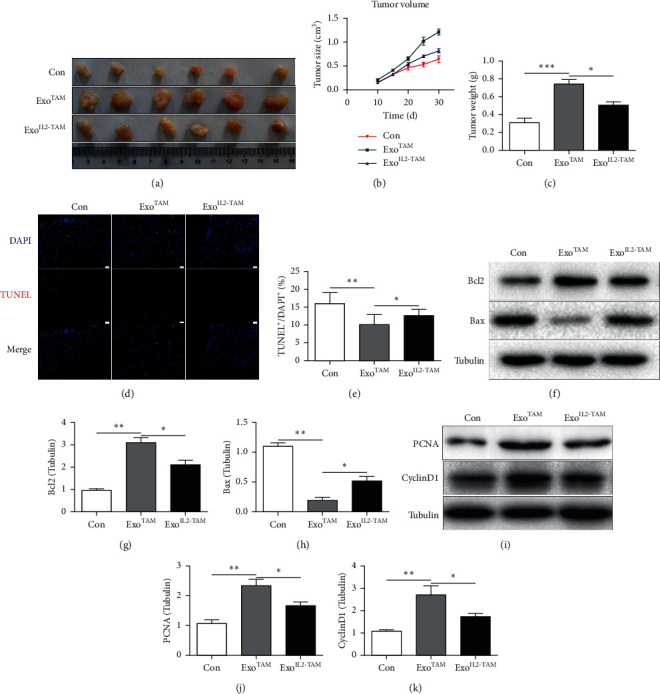
The effects of the exosomes on HCC progression *in vivo.* ((a)–(c)) The morphological characteristics of tumor xenograft, tumor size, and tumor weight (*n* = 6 per group). (d) Representative photographs of Tunel-stained cancer sections from different groups. Apoptotic nuclei were identified by Tunel staining (red) and total nuclei by DAPI (blue). Scale bar: 50 mm. (e) Percentages of Tunel-positive nuclei over total number of nuclei. (f–h) Western blot analysis of Bax and Bcl-2 expression. (i–k) Western blot analysis of PCNA and cyclin D1 expression. Error bars, SD. ^*∗*^*P* < 0.05; ^*∗∗*^*P* < 0.01; and ^*∗∗∗*^*P* < 0.001.

To test the above results *in vitro*, the *in vivo* xenograft model was used in nude mice. QJY‐7703 cells were injected subcutaneously into the flanks of nude mice with treatment next. The tumors produced by injection of QJY‐7703 in Exo^TAM^ group were significantly larger and heavier than those produced by QJY‐7703 cells alone. However, the tumors were smaller and lighter in the Exo^IL2-TAM^ group compared to the Exo^TAM^ group (Figures 4(a)–4(c)). The tumors were collected and the apoptosis of tumor tissues was detected by the TUNEL staining and western blot. The results showed that decreased positive proportion of TUNEL and Bcl-2 protein levels and increased Bax protein levels were found in the Exo^TAM^ group. However, positive proportion of TUNEL and Bcl-2 protein levels increased and Bax protein levels decreased in the Exo^IL2-TAM^ group compared to the Exo^TAM^ group (Figures 4(d)–4(h)). Meanwhile, the protein levels of PCNA and cyclin D1 to proliferation were reduced in the Exo^IL2-TAM^ group (Figures 4(i)–4(k)).

**Figure 5:**
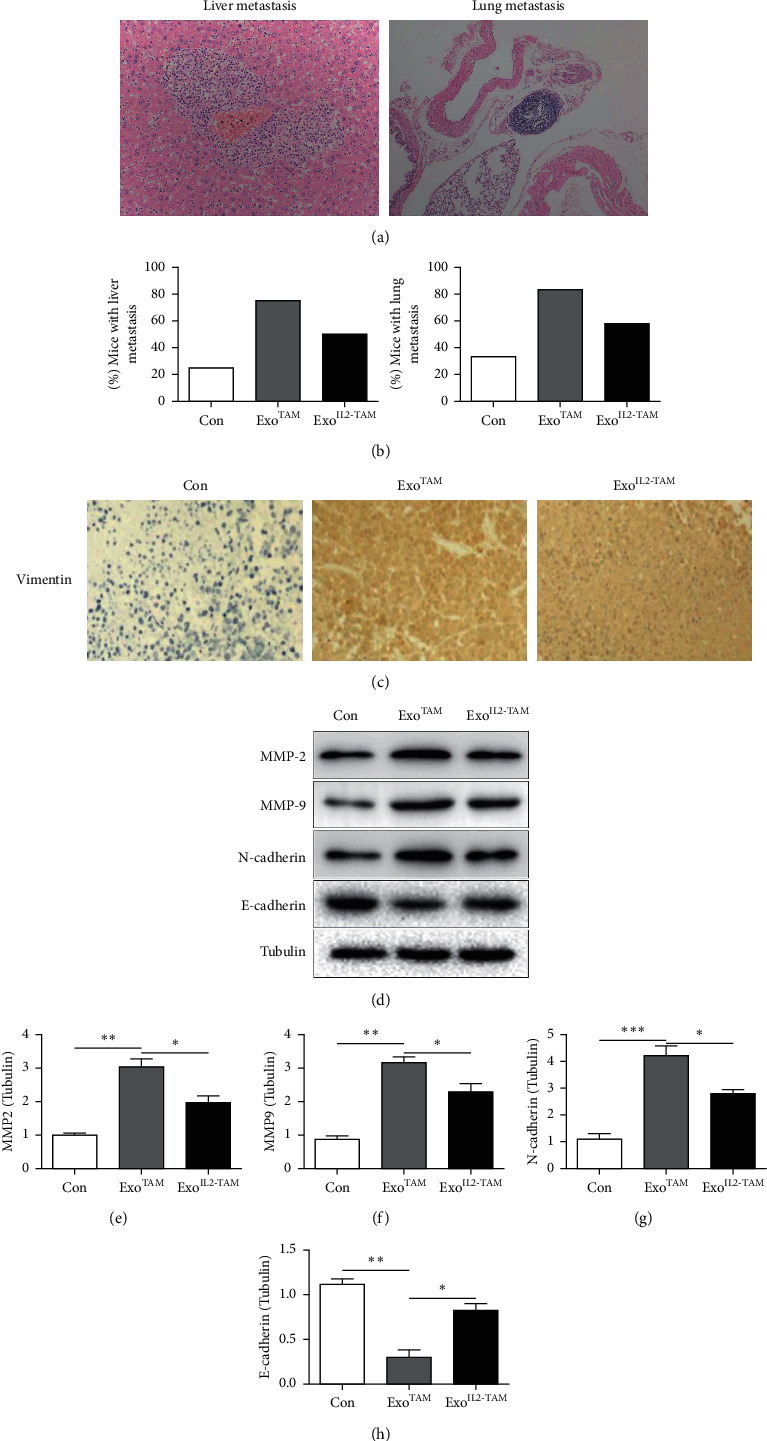
The effects of the exosomes on HCC metastasis *in vivo*. (a) Representative images of H&E-stained sections of metastatic nodules in liver and lung. (b) Percentage of mice with metastasis indicated from mice in Con, Exo^TAM^, and Exo^IL2-TAM^ groups (*n* = 12 per group). (c) Representative images of IHC staining for Vimentin in the tumors. (d–h) Western blot analysis of MMP2, MMP9, N-cadherin, and E-cadherin proteins expression. Error bars, SD. ^*∗*^*P* < 0.05; ^*∗∗*^*P* < 0.01; ^*∗∗∗*^*P* < 0.001.

To explore the effect of exosomes on tumor metastasis *in vivo*, we injected QJY‐7703 cells treated with Exo^TAM^ or with Exo^IL2-TAM^ into the nude mice via the tail vein. Quantitation of metastasis of the livers and lungs of mice in each group revealed that higher rates of hepatic and pulmonary metastases were found in the Exo^TAM^ group compared with Con group, and the rates were lower in the Exo^IL2-TAM^ group (Figures 5(a) and 5(b)). Furthermore, the IHC staining revealed that Vimentin was significantly elevated in the Exo^TAM^ group, accompanied by the increase of MMP2, MMP9, and N-cadherin and the decrease of E-cadherin. However, Vimentin, MMP2, MMP9, and N-cadherin were downregulated, and E-cadherin was upregulated in the Exo^IL2-TAM^ group compared to the Exo^TAM^ group (Figures 5(c)–5(h)).

These experiments indicated that the *in vivo* results are consistent with the *in vitro* results and IL-2 treatment ameliorates hepatocellular carcinoma development which was mediated by exosomes from TAMs.

### 3.4. The Exosomal miR-375 Was Increased from IL-2 Treated TAMs

**Figure 6:**
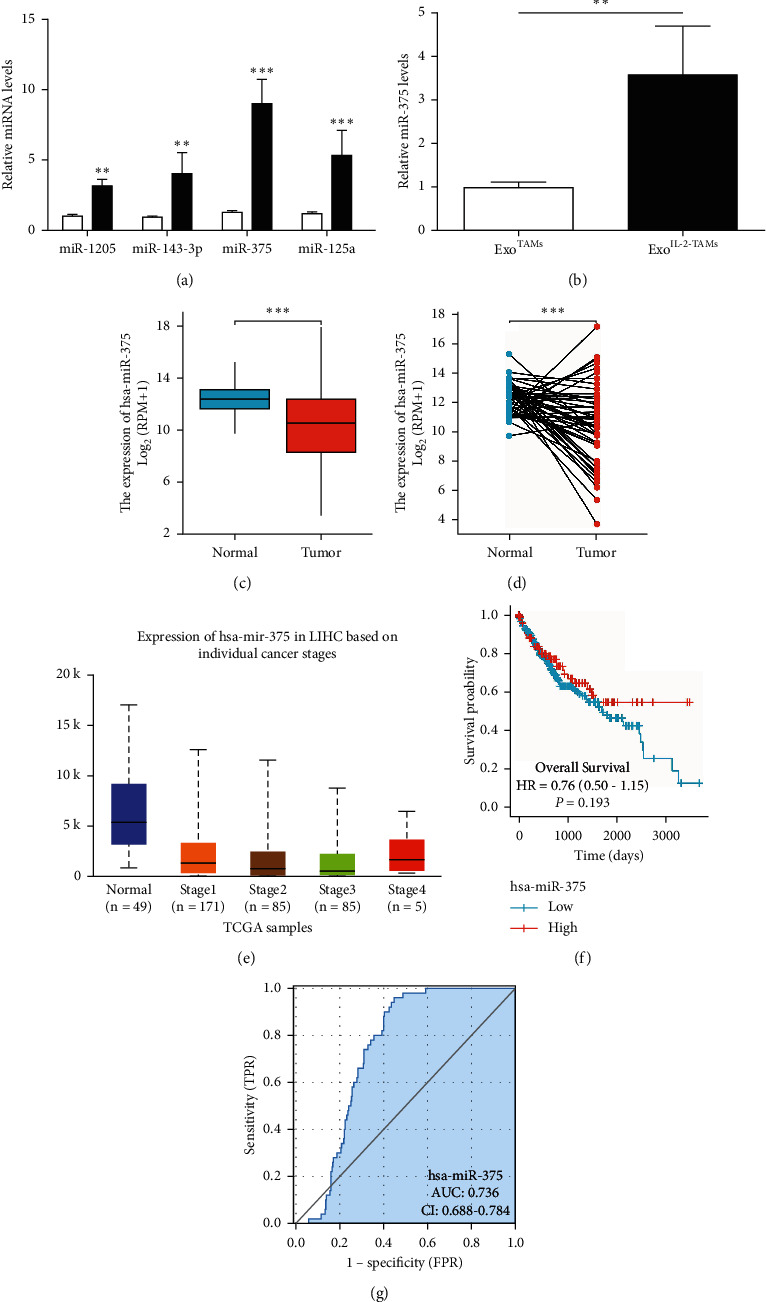
miRNAs in exosomes from IL-2-induced TAMs and in HCC patients. (a) The miRNAs expressions were detected by qRT‐PCR in exosomes from TAMs. (b) The miR-375 expressions detected by qRT‐PCR in HCC cells. (c) Nonpaired HCC tissues in TCGA database. (d) Paired HCC tissues in TCGA database. (e) HCC patients in different stages in UALCAN database. (f–g) Survival curve, ROC curve identification, and diagnosis value from TCGA database. ^*∗*^*P* < 0.05; ^*∗∗*^*P* < 0.01; ^*∗∗∗*^*P* < 0.001.

The exosomal miRNAs were regarded as an important mechanism of crosstalk between cancer cells and TAMs [19]. It was reported that some miRNAs were reduced in TAMs compared with the macrophages from normal tissues and they are able to regulate the apoptosis of tumor cells [3]. It has been well documented that miRNAs, such as miR-1205, miR-143-3p, miR-375, and miR-125a, may inhibit HCC [20–23]. As shown in Figures 6(a) and 6(b), among four miRNAs, miR-375 was obviously increased in exosomes derived from TAMs with IL-2 treatment and miR-375 was increased in Exo^IL2-TAM^ treated HCCs compared with that in Exo^IL2-TAM^ treated HCCs. We analyzed miR-375 expression in HCC patients in TCGA database and UALCAN database to explore the role of miR-375 in HCC. The results of Welch's *t*-test showed that the expression of miR-375 in Tumor group was lower than that in Normal group, and the difference between the two groups was −1.949, and the difference was statistically significant (Figure 6(c)). The paired-samples *t*-test results showed that the expression of miR-375 in Tumor groupwas higher than that in Normal group, and the difference was statistically significant (Figure 6(d)). At the same time, we can also see that the expression of miR-375 in different stages of cancer was lower than that in normal tissues (Figure 6(e)).

We can conclude that the prognosis of the low miR-375 expression group was much more severe than that of the high miR-375 expression group, suggesting that the absence of miR-375 served as unfavorable factors to the patients (Figure 6(f)). In predicting Tumor and Normal outcomes, the predictive ability of the variable hsa-miR-375 was with certain accuracy (Figure 6(g)).

## 4. Discussion

The malignant behaviors of tumor are not only determined by the characteristics of tumor cells but also affected and regulated by various components in tumor microenvironment. TAMs, as the most abundant immune cells in tumor immune microenvironment, play an important role in bridging the inflammatory mediators and tumors. Studies have shown that the degree of TAMs infiltration in HCC is negatively correlated with the prognosis of patients [24].

It was reported that most mature TAMs tend to M2 phenotype and function, including promoting angiogenesis, participating in tissue repair and reconstruction, and regulating inflammatory mediators response and adaptive immunity. In a variety of malignant solid tumors, TAMs infiltration can promote tumor growth, angiogenesis, invasion, and metastasis and resist immune damage [25].

TAMs and HCC cells interact to promote the progress of liver cancer: on the one hand, liver cancer cells secrete cytokines and chemokines, recruit macrophages to gather, and constantly adjust their own characteristics [26, 27]. On the other hand, mature TAMs participate in the progress of liver cancer through a variety of mechanisms, including promoting angiogenesis to obtain sufficient nutrients for liver cancer cells in the state of rapid growth, participating in the degradation of basement membrane and matrix remodeling to prepare for local invasion and distant metastasis of tumor, and negatively regulating the antihepatoma immune response to escape the immune system, and provide a “safe haven” for tumor development [28]. Therefore, the study of TAMs may help to find a molecular targeted drug that can effectively block the crosstalk between cancer cells and TAMs.

It is reported that exosomes mediate the intercellular information exchange which plays an important role in the occurrence and development of hepatocellular carcinoma [4–6]. Although the research on the relationship between cancers and exosomes from macrophages has become more and more popular in recent years, there is limited information on the exosomes derived from the cell lines [29–32]. Few studies have been carried out on the TAMs extracted directly from the tissues of patients [7–9]. Most of the studies on exosomes and their signaling pathways are only in the early stage, and many studies are limited to cell experiments and the results need to be further verified in animal models [31, 33]. We directly collected HCC tissues and isolated TAMs which retain some of their biological characteristics *in vivo*. More importantly, the effects of exosomes *in vivo* were explored.

Recent research has shown that IL-2 is a cytokine produced by activating T cells whose antitumor mechanism lies in stimulating and activating its effector cells and then plays an antitumor role [33, 34]. Recent studies have shown that IL-2 can promote the synthesis and secretion of IFN-*γ*, and IFN-*γ* regulates macrophage polarization to M1 type [35]. LPS induces macrophage M1 polarization and the M1 macrophages release exosomes to potentiate the anticancer efficacy [10]. However, LPS is not an approach for human therapy.

The miRNAs play a key role in the occurrence and development of human malignant tumors [36, 37]. In many malignant tumors, the expression levels of miRNA were changed to varying degrees compared with the surrounding normal tissues [11, 12]. More and more *in vitro* experiments show that the changes of miRNAs can effectively affect the proliferation and metastasis of tumor cells [19, 38]. It is reported that miR-375 inhibits HCC development and progression [21, 39, 40]. In this study, we found that miRNAs inhibiting HCC were upregulated in exosomes derived from TAMs treated with IL-2.

## 5. Conclusions

This study was the first to explore the effects of exosomes from IL-2 treated TAMs on HCC development and the possible mechanisms. We have found that exosomes derived from TAMs promote the development and progression of hepatocellular carcinoma *in vivo* and *in vitro* and these effects of exosomes were partly reversed by IL-2 treatment for TAMs. IL-2 increased the exosomal miRNAs which may be responsible for the HCC development. These findings provide a new perspective to explain the mechanism by which IL-2 inhibits HCC and provide basis for the clinical use of IL-2.

## Data Availability

The data used to support the findings of this study are available from the corresponding author upon request.
